# SNaPshot Assay for the Detection of the Most Common *CFTR* Mutations in Infertile Men

**DOI:** 10.1371/journal.pone.0112498

**Published:** 2014-11-11

**Authors:** Predrag Noveski, Svetlana Madjunkova, Marija Mircevska, Toso Plaseski, Vanja Filipovski, Dijana Plaseska-Karanfilska

**Affiliations:** 1 Macedonian Academy of Sciences and Arts, Research Center for Genetic Engineering and Biotechnology ‘Georgi D. Efremov’, Skopje, Republic of Macedonia; 2 Division of Clinical Pharmacology and Toxicology, The Hospital for Sick Children, Toronto, Ontario, Canada; 3 Faculty of Medicine, Clinic of Endocrinology and Metabolic Disorders, Skopje, Republic of Macedonia; 4 Clinical Hospital ‘Acibadem Sistina’, Skopje, Republic of Macedonia; Nanjing Medical University, China

## Abstract

Congenital bilateral absence of vas deferens (CBAVD) is the most common *CFTR*-related disorder (*CFTR*-RD) that explains about 1–2% of the male infertility cases. Controversial data have been published regarding the involvement of *CFTR* mutations in infertile men with non-obstructive azoospermia and oligozoospermia. Here, we describe single base extension (SNaPshot) assay for detection of 11 common *CFTR* mutations: F508del, G542X, N1303K, 621+1G->T, G551D, R553X, R1162X, W1282X, R117H, 2184insA and 1717-1G->A and IVS8polyT variants. The assay was validated on 50 previously genotyped samples and was used to screen a total of 369 infertile men with different impairment of spermatogenesis and 136 fertile controls. Our results show that double heterozygosity of cystic fibrosis (CF) and *CFTR*-related disorder (*CFTR*-RD) mutations are found in a high percentage (22.7%) of infertile men with obstructive azoospermia, but not in other studied groups of infertile men. The SNaPshot assay described here is an inexpensive, fast and robust method for primary screening of the most common *CFTR* mutations both in patients with classical CF and *CFTR*-RD. It can contribute to better understanding of the role of *CFTR* mutations in impaired spermatogenesis, ultimately leading to improved management of infertile men.

## Introduction

Infertility which is defined as an inability to conceive in a period of one year has prevalence of about 15% among couples of reproductive age worldwide and approximately in 50% of these cases the male factor is responsible for the failure of conception [1], [2].

The causes for male infertility can be classified as genetic and non-genetic. Chromosomal aberrations and microdeletions of the Y chromosome represent the major genetic factors of male infertility. Mutations in the cystic fibrosis transmembrane conductance regulator (*CFTR*) gene (OMIM 602421) are responsible for one of the best known autosomal genetic disorders contributing to reproductive failure [3], [4].

Mutations in *CFTR* gene are causing cystic fibrosis (CF) or related phenotypes, named *CFTR*-related disorders (*CFTR*-RD). Altered CFTR protein leads to malfunction of chloride channels and subsequent defective chloride and bicarbonate anions transport in the epithelial cells of intestine, respiratory system, pancreas, gall bladder and sweat glands. CF is a multi-system disorder that may affect normal functioning of pulmonary, pancreatic, gastro-intestinal and reproductive systems [5]. Infertility is one of the clinical manifestations of CF affecting equally both sexes with/without clinically apparent disease.

*CFTR*-RD are milder clinical entities associated with *CFTR* dysfunction, such as congenital bilateral absence of the vas deferens (CBAVD), disseminated bronchiectasis, chronic pancreatitis or chronic rhinosinusitis. CBAVD is the most common *CFTR*-RD and accounts for 1–2% of the population of infertile, but otherwise healthy, males and up to 25% of those with obstructive azoospermia [6]. Impaired CFTR protein function during the embrional stage affects the development of male reproductive system leading to improper differentiation of Wolffian ducts causing CBAVD [7].

There are more than 1900 *CFTR* mutations described to date (http://www.genet.sickkids.on.ca/StatisticsPage.html). The severity and presentation of the disease (CF or *CFTR*-RD) may depend on the type of mutation and many studies have investigated the association between the genotype and the phenotype [8], [9].

The three *CFTR* mutations most commonly associated with CBAVD are: c.1521_1523delCTT (F508del), c.1210-12T(5) (5T variant allele in intron 8) and c.350G>A (R117H) [10], [11], [12]. Most of the cases with CBAVD are usually compound heterozygous for two different alleles and the most frequent mutations involved are F508del with 5T or R117H [6], [13], [14]. The 5T allele is known to modify the expression of the R117H mutation when it is present on the same chromosome (in *cis*). R117H mutation can be found in *cis* with IVS8-5T or -7T underlying a mild CF-causing complex allele when in *cis* with IVS8-5T, or as a *CFTR*-RD mutation when in *cis* with IVS8-7T [15].

Recent studies present an evidence for involvement of CFTR protein in several pathways in the process of spermatogenesis [16], [17], [18], [19], [20], [21]. In addition, several studies have shown increased frequencies of *CFTR* mutations in infertile patients with non-obstructive azoospermia, impaired spermatogenesis and low sperm quality [22], [23], [24], [25]. However, other studies have shown no association of *CFTR* mutations with idiopathic male infertility [26], [27], [28]. Therefore, larger studies of *CFTR* mutation screening among unselected infertile males with different sperm counts may contribute to the elucidation of the role of *CFTR* gene in the impaired spermatogenesis and male infertility.

Here, we present a rapid, reliable, cost-effective and simple assay for testing of 11 *CFTR* (NM_000492.3) mutations: F508del (c.1521_1523delCTT), G542X (c.1624G>T), N1303K (c.3909C>G), 621+1G->T (c.489+1G>T), G551D (c.1652G>A), R553X (c.1657C>T), R1162X (c.3484C>T), W1282X (c.3846G>A), R117H (c.350G>A), 2184insA (c.2052_2053insA) and 1717-1G>A (c.1585-1G>A). These are the most common *CFTR* mutations overall with the highest frequencies found in Southern Europe [29] and Republic of Macedonia populations [30], [31]. Moreover the assay tests for polyT variants (5T, 7T and 9T) in intron 8 (c.1210-12T[5_9]). The methodology for screening is based on single tube multiplex PCR reaction followed by multiplex fluorescent single-nucleotide primer extension coupled with automated capillary electrophoresis which has already been successfully implemented for several other applications in our laboratory [32], [33], [34], [35].

## Materials and Methods

### Reference samples

To optimize and validate the assay genomic DNA from 25 CF patients and carriers, and from 25 normal individuals, previously genotyped using commercial genotyping kits Inno-LiPA *CFTR*19 and Inno-LiPA *CFTR*17+Tn Update Kits (Innogenetics, Ghent, Belgium) were used as the reference material. These samples were used to determine the reproducibility and performance (specificity and sensitivity) of the single-nucleotide primer extension (SNaPshot) method for detection of *CFTR* mutations.

### Patients and controls

A total of 505 DNA samples were screened for *CFTR* mutation using the newly developed *CFTR* SNaPshot assay. Briefly, DNA from peripheral blood was extracted from 260 infertile men with azoospermia (n = 79), oligozoospermia (n = 108) and normoasthenoteratozoospermia (n = 73) as well as 136 fertile controls. The assay was also used to detect *CFTR* mutations in 109DNA samples extracted from testicular biopsies (Formalin Fixed Paraffin Embedded-FFPE samples) of patients with azoospermia (n = 99) and severe oligozoospermia (n = 10).

DNA from peripheral blood was isolated using standard phenol-chloroform protocol while from FFPE samples was isolated using AllPrep DNA/RNA FFPE Kit (Qiagen, Hilden, Germany) following manufacturer’s protocol. The quality and quantity of the extracted DNA was assessed using a NanoDrop 1000 Spectrophotometer (Thermo Scientific, USA).

Clinical information and the results of spermograms including the sperm counts were available for all patients in the study. The histopathology results were available for all testicular biopsies. Based on these results 22 azoospermic patients were diagnosed with obstructive azoospermia (normal spermatogenesis on histopathology), and 87 patients with nonobstructive azoospermia or severe oligozoospermia (34 with hypospermatogenesis, 18 with Sertoly Cell Only Syndrome (SCOS), 12 with maturation arrest (MA) and 23 with atrophio/fibrosis/hyalinization of the testis).

The study was approved by the Ethical Committee of the Macedonian Academy of Sciences and Arts, and all subjects have given written informed consent for participation in the study according to Declaration of Helsinki.

### Primer design and optimization of the *CFTR* SNaPshot assay

The SNaPshot method is based on hybridization of oligonucleotide primer adjacent immediately to the variant nucleotide analyzed and extension with complementary fluorescently labeled dideoxynucleotide (ddNTP). The template for annealing and extension of a primer is previously amplified PCR fragment that contains the sequence of interest. We have amplified in one reaction, multiplex of eight different DNA fragments with length from 194 bp to 480 bp that were used for the detection of 12 *CFTR* mutations (Table 1).

**Table 1:** Primers used for PCR amplification of seven *CFTR* exons and intron 8 fragment.

Mutation analyzed^a^	Name	Sequence 5′–>3′	Exon/intron amplified(bp)^b^	Length of PCR fragment amplified in bp
621+1G->T, R117H	CFTR ex4/F	TCTTGTGTTGAAATTCTCAGGGTA	exon4 (216)	374
	CFTR ex4/R	CCAGCTCACTACCTAATTTATGACA		
delF508	CFTR ex10/F	TGAATCCTGAGCGTGATTTG	exon10 (192)	302
	CFTR ex10/R	TGGGTAGTGTGAAGGGTTCAT		
G542X, G551D, R553X	CFTR ex11/F	GCCTTTCAAATTCAGATTGAGC	exon11 (95)	288
	CFTR ex11/R	CTAGCCATAAAACCCCAGGA		
2184insA	CFTR ex13/F	TGCAATAAAACATTAACAAAATGC	exon13 (724)	480
	CFTR ex13/R	GGGAGTCTTTTGCACAATGG		
R1162X	CFTR ex19/F	TGTGAAATTGTCTGCCATTCTT	exon19 (249)	369
	CFTR ex19/R	TGCTTCAGGCTACTGGGATT		
W1282X	CFTR ex20/F	CTGAATTATGTTTATGGCATGG	exon20 (156)	249
	CFTR ex20/R	TTTTTCTGGCTAAGTCCTTTTG		
N1303K	CFTR ex21/F	TGATGGTAAGTACATGGGTGTTTC	exon21 (90)	257
	CFTR ex21/R	CCCCTTTCA AAATCATTTCAG		
IVS8-5T/7T/9T	CFTR intron 8/F	GGCCATGTGCTTTTCAAACT	intron8 (194)	194
	CFTR intron 8/R	AAGAAGAGGCTGTCATCACCA		

a Legacy name.

b The exon/intron numbering is based on legacy exon intron nomenclature (http://www.genet.sickkids.on.ca/).

The design and optimization of the multiplex PCR (Table 1) and extension primers mixes (Table 2) were performed using Primer3 software [36], while the specificity was confirmed using BLAST software (http://blast.ncbi.nlm.nih.gov). Primers were designed not to include regions with known SNPs as polymorphisms and mutations within primer-annealing regions of the template, especially those close to the 3′ end of a primer, could create imperfections in base paring and decrease primer extension efficiency.

**Table 2:** *CFTR* multiplex SNaPshot primer extension mix with primer orientation, size and concentrations.

CFTR mutation	cDNA name according toHGVS (ref. seq. NM_000492.3)	Sequence (5′->3′)	Orientation	SNaPshotResult(normal/mutantallele)	Size ofextendedfragment inbase pairs(normal allele/mutant allele)^a^	Concentration in mix (µM)^b^
G542X	c.1624G>T	CAGTGTGATTCCACCTTCTC	Reverse	**C/A**	(24.9/25.9)	3
N1303K	c.3909C>G	CCCACTGTTCATAGGGATCCAA	Reverse	**G/C**	(26.3/26.9)	5
F508del	c.1521_1523delCTT	CCCCTGGCACCATTAAAGAAAATATCAT	Forward	**C/T**	(29.6/31.0)	1
R117H	c.350G>A	15(C)GGATAACAAGGAGGAAC	Forward	**G/A**	(33.6/35.3)	7
IVS8-5T/7T/9T	c.1210-12T[5_9]	TGTGTGTGTGTGTGTGTGTTTTT	Forward	**A/T**	5T - 32.3 7T,9T - 33.4	1
621+1G->T	c.489+1G>T	CCCTAGCTATGTTTAGTTTGATTTATAAGAAG	Forward	**G/T**	(37.2/38.2)	5
IVS8-7T/9T	c.1210-12T[7_9]	14(C)GTGTGTGTGTGTGTGTGTTTTTTT	Forward	**A/T**	7T - 44.0 9T - 44.9	2
2184insA	c.2052_2053insA	13(C)GTCTCCTGGACAGAAACAAAAAAA	Forward	**C/A**	(38.7/39.7)	8
1717-1 G->A	c.1585-1G>A	9(C)GACTCTCTAATTTTCTATTTTTGGTAATA	Forward	**G/A**	(41.3/41.7)	2
G551D	c.1652G>A	21(C)TGGAATCACACTGAGTGGAG	Forward	**G/A**	(43.4/43.9)	4
R553X	c.1657C>T	24(C)AATCACACTGAGTGGAGGTCAA	Forward	**C/T**	(46.2/47.2)	2
W1282X	c.3846G>A	28(C)GGATTCAATAACTTTGCAACAGTG	Forward	**G/A**	(51.6/52.6)	1
R1162X	c.3484C>T	29(C)ATTTCAGATGCGATCTGTGAGC	Forward	**C/T**	(51.0/52.0)	4

a Data generated on ABI PRISM 3130 Genetic Analyzer with POP-4 polymer, 36-cm capillary array and sized against GeneScan-120 LIZ size standard.

b Concentration in the SNaPshot extension primer mix adjusted to produce relatively equal peak heights.

Electrophoretic mobility and separation of extended oligonucleotides depends not only on their size but also on their sequence and type of label (extended ddNTP). For oligonucleotides that had same electrophoretic mobility and/or size overlap, we either redesigned the sequence or added a polyC tail on the 5′ end on one of the ambiguous primers (Table 2). Also, we further optimized the SNaPshot multiplex by testing each of the extension primers for *CFTR* gene variants individually and in multiplex on samples with known *CFTR* genotypes. For this purpose we used 50 samples with 14 different *CFTR* genotypes (Table 3).

**Table 3:** *CFTR* genotypes in 50 DNA samples used for validation of the SNaPshot method.

Genotype withlegacy namefrom Inno Lipa kits	cDNA name according toHGVS (reference sequenceNM_000492.3)	Number ofpatientstested	Concordance with *CFTR* SNaPshot assay
[−]/[−]	[ = ];[ = ]	25	100%
F508del/F508del	c.[1521_1523delCTT];[1521_1523delCTT]	9	100%
F508del/[−]	c.[1521_1523delCTT];[ = ]	3	100%
G542X/[−]	c.[1624G>T];[ = ]	2	100%
621+1G>T/[−]	c.[489+1G>T];[ = ]	2	100%
G542X/F508del	c.[1624G>T];[1521_1523delCTT]	1	100%
R117H/F508del	c.[350G>A];[1521_1523delCTT]	1	100%
2184insA/F508del	c.[2052_2053insA];[1521 _1523delCTT]	1	100%
W1282X/[−]	c.[3846G>A];[ = ]	1	100%
R1162X/[−]	c.[3484C>T];[ = ]	1	100%
R553X/R553X	c.[1657C>T];[1657C>T]	1	100%
N1303K/[−]	c.[3909C>G];[ = ]	1	100%
1717-1 G>A/[−]	c.[1585-1G>A];[ = ]	1	100%
G551D	c.[1652G>A];[ = ]	1	100%

Note: [−] means no CF mutation detected on single chromosome.

### PCR conditions and capillary electrophoresis

PCR reaction mix contained 1X reaction buffer B2, 2.5 mM MgCl_2_, 200 µM each dNTP, 100 nM each primer, 1X Solution S, 1 U HOT FIREPol DNA polymerase (Solis BioDyne, Tartu, Estonia) and 100 ng genomic DNA. Cycling conditions were: initial ‘hot-start’ denaturation at 95°C for 10 min followed by 35 cycles of 95°C for 1 min, 55°C for 1 min, 72°C for 1 min 30 s, and a final extension at 72°C for 10 min. To remove unincorporated nucleotides and excess primers 1 ul of PCR product was cleaned up with 1 ul of ExoSAP-IT (Affymetrix, Inc., Santa Clara, California, USA) for 60 min on 37°C followed by enzyme heat inactivation for 20 min on 86°C. Subsequently, 2 ul of purified PCR multiplex mix was combined with 1 ul extension primer mix and 1 ul of SNaPshot Multiplex Ready Reaction Mix (Life Technologies, Carlsbad, California, USA).

**Figure 1:**
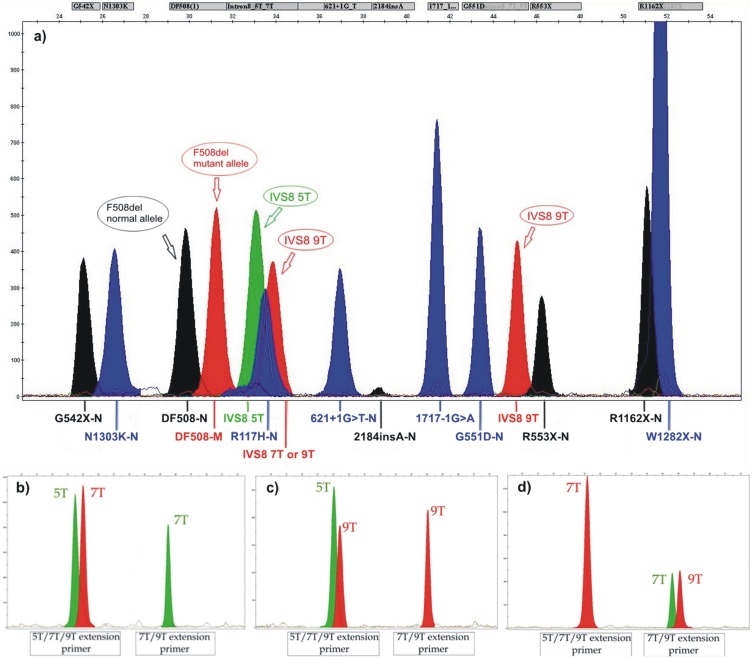
Representative electrophoreograms of *CFTR* SNaPshot multiplex assay. a) Electrophoreogram of *CFTR* SNaPshot multiplex assay for detection of 12 common *CFTR* gene mutations showing a compound heterozygote for F508del/IVS8-5T. The fluorescence intensity is represented on the Y axis of the electrophoreogram and the fragments’ size on the X axis. N = normal allele (wild type) M = mutant allele; b-d) Electrophoreograms of samples with different IVS8polyT genotypes: 5T/7T (b), 5T/9T (c) and 7T/9T (d).

Primer extension was performed on a thermal cycler for 25 cycles with the following cycling conditions: 96°C for 10 sec, 50°C for 5 sec and 60°C for 30 sec. The unincorporated ddNTPs were removed from the reaction mix by the use of 1 U shrimp alkaline phosphatase (Affymetrix, Inc., Santa Clara, California, USA) for 60 min on 37°C, followed by enzyme heat inactivation for 15 min on 65°C. A 1 ul of cleaned products was combined with 12 ul HiDi formamide (Life Technologies) and 0.5 ul GeneScan-120LIZ size standard (Life Technologies), heat denatured at 95°C for 5 min then transferred on ice for 2 minutes and loaded onto an ABI PRISM 3130 Genetic Analyzer (Life Technologies). Capillary electrophoresis was conducted following manufacturer instructions on a 36 cm length capillary and POP-4 polymer. Data were analyzed using GeneMapper analysis software version 4.0. A representative electrophoreogram is given in Figure 1a.

### Determination of Intron 8 polyT strand (5T/7T/9T) with single base extension approach

**Figure 2:**
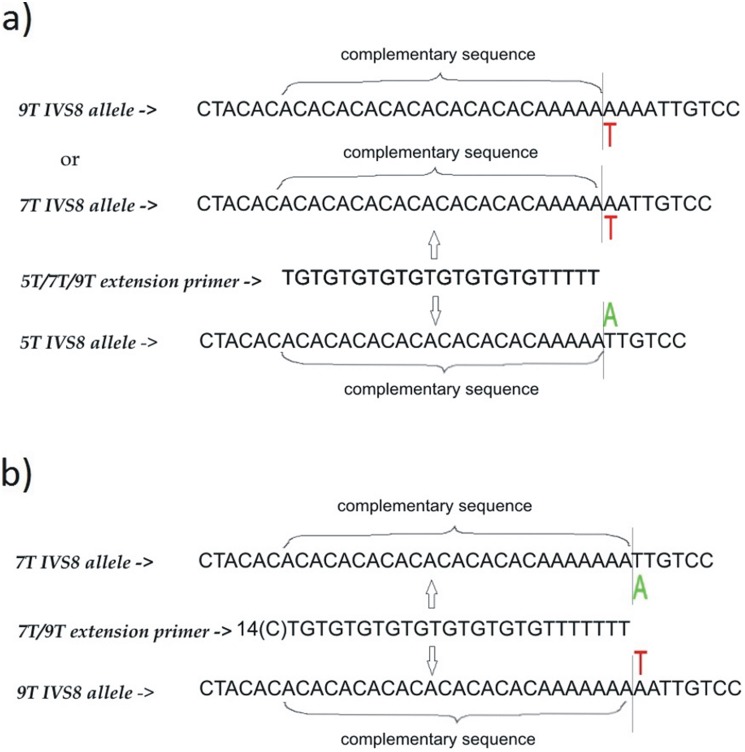
Strategy for detection of IVS8polyT alleles with single base extension aproach. a) ‘5T/7T/9T extension primer’ in the presence of 5T allele is extended with dideoxyadenine while in the presence of 7T/9T alleles is extended with dideoxythymidine; b) ‘7T/9T extension primer’ in the presence of 7T allele is extended with dideoxyadenine while in the presence of 9T allele is extended with dideoxythymidine. Results of the two primers extension reactions give the final genotype. Extension primer sequences are given in 5′->3′ orientation, while the alleles represent the complementary (minus) strand.

To determine the length of intron 8 polyT strand we designed a set of PCR primers (forward and reverse) amplifying the region containing the T-alleles and two extension primers for subsequent single base extension analysis. One of the extension primers, designated as ‘5T/7T/9T extension primer’ can detect any of the polyT alleles (5T, 7T and 9T) while the other one, designated as ‘7T/9T extension primer’ can detect only 7T and 9T alleles (Figure 2). The ‘5T/7T/9T extension primer’ is discriminating the 5T allele from 7T/9T alleles. This primer has 5 thymidine residues at the 3′ end and in the presence of 5T allele it is extended with dideoxyadenosine (green peak), while in the presence of 7T or 9T alleles it is extended with dideoxythymidine (red peak on the electrophoreogram). The ‘7T/9T extension primer’ is further discriminating the 7T and 9T alleles. This primer has 7 thymidine residues at the 3′ end and in the presence of 7T allele it is extended with dideoxyadenosine (green peak) while in the presence of 9T allele it is extended with dideoxythymidine (red peak). Electrophoreograms of samples with three different IVS8polyT genotypes (5T/7T, 5T/9T and 7T/9T) are given in Figure 1b, 1c and 1d.

### Determination of the number of TG repeats adjacent to the IVS8polyT alleles

The TG number immediately upstream of the IVS8polyT allele was determined by multiplex allele-specific fluorescent PCR in 25 men who carried IVS8-5T allele (5 compound heterozygotes for a CF mutation and IVS8-5T and 20 IVS8-5T heterozygotes with no other CF mutation) following the procedure described previously [37].

### Statistical analysis

Statistical non-parametric tests for categorical variables (Pearson chi-square or Fischer exact test) were used when appropriate. The level of significance was set at p<0.05 and data analysis was conducted using Statistical Package for Social Sciences Version 19 (SPSS, Chicago, IL, USA).

## Results

The validation of the *CFTR* SNaPshot assay carried out on 50 previously genotyped DNA samples demonstrated that it could accurately detect the 11 tested *CFTR* mutations and IVS8polyT alleles. Feasibility and reproducibility of the assay was assessed first by testing the normal samples and the specificity and sensitivity was further tested by analyzing the CF patients and CF carriers. All extension primers multiplexed in one *CFTR* SNaPshot mix, had electrophoretic mobility and fragment sizes in agreement with those obtained when each was tested individually without any nonspecific peaks or ambiguous results. Our results showed 100% concordance with the independently determined genotypes attesting that the new assay was highly accurate (Table 3).

The *CFTR* SNaPshot assay was used for detection of 12 different *CFTR* mutations in a total of 505 men, of whom 136 were fertile controls with proven paternity by DNA analysis. The infertile men consisted of 260 men who were divided into three groups according to the sperm counts, i.e. azoospermia (n = 79), oligozoospermia (n = 108), normoasthenoteratozoospermia (n = 73) and a group of 109 men who were divided into two groups according to the histopathological results of the testicular biopsies, i.e. obstructive azoospermia (n = 22) and non-obstructive azoospermia/severe oligozoospermia (n = 87).

Five patients (22.7%) of the 22 patients with obstructive azoospermia had F508del/IVS8-5T genotype consistent with a CBAVD diagnosis, and two patients were heterozygotes for F508del mutation. In the group of 87 patients with histopathology consistent with nonobstructive azoospermia/severe oligozoospermia only one patient with F508del/IVS8-5T genotype was detected who presented with azoospermia and histopathological result of testicular atrophy. Two patients were F508del heterozygotes and two patients were heterozygotes for 5T allele variant (Table 4).

**Table 4:** Distribution of *CFTR* and IVS8polyT genotypes in the two groups of patients divided according to the histopathological results: obstructive azoospermia and nonobstructive azoospermia/oligozoospermia.

Histopatological diagnosis	*CFTR* genotype	Intron8 polyT genotype	N	%
**Obstructive azoospermia**	delF508/[−]	5T/9T	5	22.7%
	delF508/[−]	7T/9T	2	9.1%
	[−]/[−]	7T/7T	13	59.1%
	[−]/[−]	7T/9T	2	9.1%
	Total		22	100.0%
**Nonobstructive azoospermia +** **severe oligozoospermia**	delF508/[−]	5T/9T	1	1.1%
	delF508/[−]	7T/9T	3	3.4%
	[−]/[−]	5T/7T	2	2.3%
	[−]/[−]	7T/7T	60	69.0%
	[−]/[−]	7T/9T	21	24.1%
	Total		87	100.0%

[−] means no CF mutation detected on single chromosome.

The distribution of the *CFTR* and IVS8polyT genotypes in the group of 260 infertile patients categorized in accordance to the sperm counts is shown in Table 5. Nine azoospermic patients were heterozygous for 5T allele variant. Among the oligozoospermic men five patients carried CF mutation (3 with F508del and 2 with G542X) but one of them also carried the *CFTR*-RD mutation, i.e. 5T allele. This patient manifested with mild oligozoospermia with sperm concentration of 11×10^6^/ml additionally, four patients were heterozygous for 5T allele variant. Three normoasthenoteratozoospermic men were carriers of F508del mutation and additional 6 were carriers of 5T allele. Among the fertile controls, two men carried F508del mutation and additional 9 were carriers of 5T allele.

**Table 5:** Distribution of *CFTR* and IVS8polyT genotypes in infertile men with different sperm counts and fertile controls.

Patients	*CFTR* genotype	Intron8 polyT genotype	N	%
**Azoospermia**	[−]/[−]	5T/7T	8	10.1%
	[−]/[−]	5T/9T	1	1.3%
	[−]/[−]	7T/7T	56	70.9%
	[−]/[−]	7T/9T	14	17.7%
	Total		79	100.0%
**Oligozoospermia**	delF508/[−]	7T/9T	2	1.9%
	delF508/[−]	9T/9T	1	0.9%
	G542X/[−]	5T/9T	1	0.9%
	G542X/[−]	7T/9T	1	0.9%
	[−]/[−]	5T/7T	4	3.7%
	[−]/[−]	7T/7T	79	73.1%
	[−]/[−]	7T/9T	20	18.5%
	Total		108	100.0%
**Normoasthenoteratozoospermia**	delF508/[−]	7T/9T	1	1.4%
	delF508/[−]	9T/9T	2	2.7%
	[−]/[−]	5T/7T	6	8.2%
	[−]/[−]	7T/7T	49	67.1%
	[−]/[−]	7T/9T	14	19.2%
	[−]/[−]	9T/9T	1	1.4%
	Total		73	100.0%
**Fertile Controls**	delF508/[−]	7T/9T	2	1.5%
	[−]/[−]	5T/7T	8	5.9%
	[−]/[−]	5T/9T	1	0.7%
	[−]/[−]	7T/7T	96	70.6%
	[−]/[−]	7T/9T	27	19.9%
	[−]/[−]	9T/9T	2	1.5%
	Total		136	100.0%

Note: [−] means no CF mutation detected on single chromosome.

Table 6 summarizes the results of the *CFTR* genotypes among the five groups of infertile men in comparison to the fertile controls. Compound heterozygosity for CF mutation (F508del or G542X) and *CFTR*-RD mutation (IVS8-5T) was present in higher frequency among patients with obstructive azoospermia (22.7%), when compared to the fertile controls (0%) p = 3.42×10^−5^ and all other groups of infertile men: non-obstructive azoospermia/severe oligozoospermia (1.14%, p = 1.16×10^−3^), azoospermia (0%, p = 3.32×10^−4^), oligozoospermia (0.92%, p = 4.89×10^−4^) and normoasthenoteratozoospermia (0%, p = 4.54×10^−4^). Although the heterozygosity for CF mutation was present with higher frequency among patients with obstructive azoospermia than among fertile controls and all other groups of infertile men, these differences were not statistically significant. There was also no statistically significant difference in the distribution of the IVS8-5T carriers among the studied groups.

**Table 6:** Distribution of genotypes (compound heterozygous, heterozygous or normal) in patients with and without histopatological data.

Genotypes	Histopatological data		No hisptopatological data			
	Obstructive azoospermia (n = 22)	Non-obstructive azoospermia + severe oligozoospermia (n = 87)	Azoospermia (n = 79)	Oligozoospermia (n = 108)	Normoasthenoteratozoospermia (n = 73)	Fertile controls (n = 136)
**CF mutation/5T**	5 (22.7%)	1 (1.14%)	0	1 (0.92%)	0	0
**CF mutation/[−]**	2 (9.09%)	3 (3.44%)	0	4 (3.70%)	3 (4.10%)	2 (1.47%)
**5T/[−]**	0	2 (2.29%)	9 (11.39%)	4 (3.70%)	6 (8.21%)	9 (6.61%)
**Total**	7 (31.8%)	6 (6.89%)	9 (11.39%)	9 (8.33%)	9 (12.33%)	11 (8.08%)
**[−]/[−]**	15 (68.20%)	81 (93.11%)	70 (88.61%)	93 (86.11%)	64 (87.67%)	125 (91.92%)

Note: [−] means no CF mutation detected on single chromosome.

## Discussion

Our study has proven that the new SNaPshot assay developed to detect 12 different *CFTR* mutations (11 mutations and IVS8-5T allele) can be accurately used for screening and diagnosis of *CFTR* mutations among different groups of affected patients including infertile men. Cystic fibrosis has different degrees of severity and clinical manifestations depending on the type of mutations (severe or mild) and their combination (*cis* or *trans* positions on chromosomes) which could result in ‘classical’ or ‘atypical’ form of the disease. The ‘atypical CF’ or *CFTR*-RD is mainly organ oriented. CBAVD is the most common *CFTR*-RD, which is usually associated with the presence of one severe mutation (most commonly F508del) and one mild mutation (most commonly R117H or IVS8-5T) or two mild mutations.

The intron 8 polyT tract, adjacent to the *CFTR* exon 9 splice acceptor site contains 5, 7 or 9 thymidine bases. This variation affects the efficiency of the splice site, with the 5T variant being associated with the least efficient splicing of exon 9 and subsequently the highest levels of mRNA lacking exon 9, leading to reduced production of functional CFTR protein. It is estimated that the 5T allele is found in approximately 5% of alleles in the general population. It has been reported that exon 9 skipping on IVS8-5T alleles can be further modulated by the number of adjacent TG repeats [38]. It was shown that most of the patients with CBAVD carry 12 or 13 TG repeats, while most of the normal individuals have 11 TG repeats immediately upstream of the IVS8-5T allele [39]. Apart from these three mutations there are reports for other mutations found in men with CBVAD, most frequently in combination with the three previously mentioned [13].

Several studies have reported an increased frequency of *CFTR* gene mutations in men with non-obstructive oligozoospermia and impaired spermatogenesis [22], [23], [24], [25]. An evidence for involvement of CFTR protein in several pathways in the process of spermatogenesis has also been suggested [16], [17], [18], [19], [20], [21].

With an aim to contribute to the elucidation of the role of *CFTR* gene in the impaired spermatogenesis and male infertility, we designed a screening assay for 11 most common *CFTR* gene mutations and IVS8polyT variant, based on primer extension with fluorescent dideoxynucleotides and subsequent detection on capillary electrophoresis. The assay was validated with samples previously genotyped with commercial kits.

This method was used to screen for the presence of the most common CF and *CFTR*-RD mutations among 369 infertile males divided in two groups (109 patients with and 260 patients without histopathological data) and in 136 fertile controls. Our results show that *CFTR* mutations are found in a high percentage of infertile men with obstructive azoospermia (5/22 or 22.7%), which is consistent with the data from other studies [40]. All 5 patients were double heterozygotes for a CF F508del mutation and a *CFTR*-RD IVS8-5T variant. Although the double heterozygosity in the patients with F508del/IVS8-5T genotype was not confirmed by studies of the patient’s parents, 5T was inferred to be in *trans* with F508del, since this mutation has never been found in linkage disequilibrium with 5T [39].

We could not demonstrate an increased frequency of double heterozygosity of *CFTR* mutations among other studied groups of infertile men. We have genotyped one patient to have F508del/IVS8-5T genotype, with histopathological diagnosis of testicular atrophy and one oligozoospermic patient with G542X/IVS8-5T genotype. The analysis of the TG repeats adjacent to the 5T showed 11TG repeats in the patient with testicular atrophy and 12TG repeats in the oligozoospermic patient. These results imply that F508del/IVS8-11TG-5T genotype might not be a cause of the infertility in the first patient, while G542X/IVS8-12TG-5T genotype is most probably a cause of infertility in the second patient. Similar to F508del, G542X has never been found in linkage disequilibrium with IVS8-5T [39].

The analysis for determination of TG numbers were also performed in 3 of the 5 obstructive azoospermic patients with F508del/IVS8-5T genotypes and in 20 men with 5T allele and no other *CFTR* mutation. Two men with F508del/IVS8-5T genotype had 12, while one had 13TG repeats. Fourteen of the 20 men with 5T allele had 11TG repeats, while 6 had 12TG repeats. All fertile controls analyzed (n = 4) had 11TG repeats. Three of the 6 men with 12TG-5T were azoospermic, one was oligozoospermic and two were normoasthenoteratozoospermic. It might be interesting to search for other CF mutations in the azoospermic patients with 12TG-5T allele, especially if they have CBAVD. Unfortunately, we do not have any other clinical or laboratory data on these patients apart from the sperm counts to confirm that they have obstructive azoospermia and CBAVD.

The heterozygosity for CF mutation was highest among patients with obstructive azoospermia (9.09%). With the exception of the patients with azoospermia where no CF mutation was detected in all other groups of infertile men the CF heterozigosity was higher (ranging from 3.44 to 4.10%) when compared to the fertile controls (1.47%). Although these differences were not statistically significant, the higher percentage of CF mutations among infertile patients, especially those with obstructive azoospermia might imply a presence of some other mild *CFTR*-RD variant in these patients that was not investigated by our screening assay.

Assisted reproduction techniques that are rapidly evolving are increasing the chances for reproduction among infertile men with *CFTR* mutations along with the increased risk of passing the mutation to the offspring. Our assay may serve as primary screening tool for the most common *CFTR* mutations. In case a single CF or *CFTR*-RD mutation is detected the search for second mutation should be extended with sequencing of all exons and splicing sites or appropriate molecular diagnostic techniques for detecting larger genomic rearrangements. This is giving the importance of this test in the view of proper risk calculations for prospective parents and adequate genetic counseling.

Previously, one study used the SNaPshot method, but only for the detection of paternally-inherited fetal mutations in maternal plasma in three families at risk for a child with CF [41]. Here, we describe the first fully optimized and validated method for screening of several common *CFTR* mutations, based on multiplex single nucleotide base extension using fluorescently labeled dideoxynucleotides. This method utilizes the use of Genetic Analyzers which are becoming standard equipment in most molecular diagnostic laboratories. The multiplex SNaPshot assay for *CFTR* mutation detection is a robust, inexpensive and fast method with a minimum hands-on time suited for screening of a large number of samples. It can be reliably used as a diagnostic and screening test both in patients with classical CF and atypical CF or *CFTR* related disorders.

## References

[1] Poongothai J, Gopenath TS, Manonayaki S (2009) Genetics of human male infertility. Singapore Med J 50: 336–347. 19421675

[2] Kolettis PN (2003) Evaluation of the subfertile man. Am Fam Physician 67: 2165–2172. 12776966

[3] Chan HC, Ruan YC, He Q, Chen MH, Chen H, et al (2009) The cystic fibrosis transmembrane conductance regulator in reproductive health and disease. J Physiol 587: 2187–2195. 19015188 10.1113/jphysiol.2008.164970 PMC2697292

[4] Visser L, Repping S (2010) Unravelling the genetics of spermatogenic failure. Reproduction 139: 303–307. 19776097 10.1530/REP-09-0229

[5] Rowntree RK, Harris A (2003) The phenotypic consequences of CFTR mutations. Ann Hum Genet 67: 471–485. 12940920 10.1046/j.1469-1809.2003.00028.x

[6] Yu J, Chen Z, Ni Y, Li Z (2012) CFTR mutations in men with congenital bilateral absence of the vas deferens (CBAVD): a systemic review and meta-analysis. Hum Reprod 27: 25–35. 22081250 10.1093/humrep/der377

[7] Wong PY (1998) CFTR gene and male fertility. Mol Hum Reprod 4: 107–110. 9542966 10.1093/molehr/4.2.107

[8] Zielenski J, Tsui LC (1995) Cystic fibrosis: genotypic and phenotypic variations. Annu Rev Genet 29: 777–807. 8825494 10.1146/annurev.ge.29.120195.004021

[9] Welsh MJ, Smith AE (1993) Molecular mechanisms of CFTR chloride channel dysfunction in cystic fibrosis. Cell 73: 1251–1254. 7686820 10.1016/0092-8674(93)90353-r

[10] Claustres M (2005) Molecular pathology of the CFTR locus in male infertility. Reprod Biomed Online 10: 14–41. 15705292 10.1016/s1472-6483(10)60801-2

[11] Dork T, Dworniczak B, Aulehla-Scholz C, Wieczorek D, Bohm I, et al (1997) Distinct spectrum of CFTR gene mutations in congenital absence of vas deferens. Hum Genet 100: 365–377. 9272157 10.1007/s004390050518

[12] Casals T, Bassas L, Egozcue S, Ramos MD, Gimenez J, et al (2000) Heterogeneity for mutations in the CFTR gene and clinical correlations in patients with congenital absence of the vas deferens. Hum Reprod 15: 1476–1483. 10875853 10.1093/humrep/15.7.1476

[13] De Braekeleer M, Ferec C (1996) Mutations in the cystic fibrosis gene in men with congenital bilateral absence of the vas deferens. Mol Hum Reprod 2: 669–677. 9239681 10.1093/molehr/2.9.669

[14] Claustres M, Guittard C, Bozon D, Chevalier F, Verlingue C, et al (2000) Spectrum of CFTR mutations in cystic fibrosis and in congenital absence of the vas deferens in France. Hum Mutat 16: 143–156. 10923036 10.1002/1098-1004(200008)16:2<143::AID-HUMU7>3.0.CO;2-J

[15] Dequeker E, Stuhrmann M, Morris MA, Casals T, Castellani C, et al (2009) Best practice guidelines for molecular genetic diagnosis of cystic fibrosis and CFTR-related disorders–updated European recommendations. Eur J Hum Genet 17: 51–65. 18685558 10.1038/ejhg.2008.136 PMC2985951

[16] Xu WM, Chen J, Chen H, Diao RY, Fok KL, et al (2011) Defective CFTR-dependent CREB activation results in impaired spermatogenesis and azoospermia. PLoS One 6: e19120. 21625623 10.1371/journal.pone.0019120 PMC3090391

[17] Ruan YC, Shum WW, Belleannee C, Da Silva N, Breton S (2012) ATP secretion in the male reproductive tract: essential role of CFTR. J Physiol 590: 4209–4222. 22711960 10.1113/jphysiol.2012.230581 PMC3473280

[18] Chen H, Ruan YC, Xu WM, Chen J, Chan HC (2012) Regulation of male fertility by CFTR and implications in male infertility. Hum Reprod Update 18: 703–713. 22709980 10.1093/humupd/dms027

[19] Li CY, Jiang LY, Chen WY, Li K, Sheng HQ, et al (2010) CFTR is essential for sperm fertilizing capacity and is correlated with sperm quality in humans. Hum Reprod 25: 317–327. 19923167 10.1093/humrep/dep406

[20] Chen J, Fok KL, Chen H, Zhang XH, Xu WM, et al (2012) Cryptorchidism-induced CFTR down-regulation results in disruption of testicular tight junctions through up-regulation of NF-kappaB/COX-2/PGE2. Hum Reprod 27: 2585–2597. 22777528 10.1093/humrep/des254

[21] Xu WM, Shi QX, Chen WY, Zhou CX, Ni Y, et al (2007) Cystic fibrosis transmembrane conductance regulator is vital to sperm fertilizing capacity and male fertility. Proc Natl Acad Sci U S A 104: 9816–9821. 17519339 10.1073/pnas.0609253104 PMC1887595

[22] van der Ven K, Messer L, van der Ven H, Jeyendran RS, Ober C (1996) Cystic fibrosis mutation screening in healthy men with reduced sperm quality. Hum Reprod 11: 513–517. 8671256 10.1093/humrep/11.3.513

[23] Schulz S, Jakubiczka S, Kropf S, Nickel I, Muschke P, et al (2006) Increased frequency of cystic fibrosis transmembrane conductance regulator gene mutations in infertile males. Fertil Steril 85: 135–138. 16412743 10.1016/j.fertnstert.2005.07.1282

[24] Jakubiczka S, Bettecken T, Stumm M, Nickel I, Musebeck J, et al (1999) Frequency of CFTR gene mutations in males participating in an ICSI programme. Hum Reprod 14: 1833–1834. 10402399 10.1093/humrep/14.7.1833

[25] Yu J, Chen Z, Zhang T, Li Z, Ni Y (2011) Association of genetic variants in CFTR gene, IVS8 c.1210-12T[5_9] and c.1210-35_1210-12GT[8_12], with spermatogenetic failure: case-control study and meta-analysis. Mol Hum Reprod 17: 594–603. 21427159 10.1093/molehr/gar019

[26] Tuerlings JH, Mol B, Kremer JA, Looman M, Meuleman EJ, et al (1998) Mutation frequency of cystic fibrosis transmembrane regulator is not increased in oligozoospermic male candidates for intracytoplasmic sperm injection. Fertil Steril 69: 899–903. 9591500 10.1016/s0015-0282(98)00050-8

[27] Pallares-Ruiz N, Carles S, Des Georges M, Guittard C, Arnal F, et al (1999) Complete mutational screening of the cystic fibrosis transmembrane conductance regulator gene: cystic fibrosis mutations are not involved in healthy men with reduced sperm quality. Hum Reprod 14: 3035–3040. 10601093 10.1093/humrep/14.12.3035

[28] Mak V, Zielenski J, Tsui LC, Durie P, Zini A, et al (2000) Cystic fibrosis gene mutations and infertile men with primary testicular failure. Hum Reprod 15: 436–439. 10655318 10.1093/humrep/15.2.436

[29] CFGAC (1994) Population variation of common cystic fibrosis mutations. The Cystic Fibrosis Genetic Analysis Consortium. Hum Mutat 4: 167–177. 7530552 10.1002/humu.1380040302

[30] Petreska L, Koceva S, Plaseska D, Chernick M, Gordova-Muratovska A, et al (1998) Molecular basis of cystic fibrosis in the Republic of Macedonia. Clin Genet 54: 203–209. 9788722 10.1111/j.1399-0004.1998.tb04285.x

[31] Koceva S, Plaseska-Karanfilska D, Fustic-Naceva S, Caparevska M, Efremov G (2001) Cystic Fibrosis In Macedonia: An Update. Balkan Journal of Medical Genetics 4: 47–51.

[32] Noveski P, Trivodalieva S, Efremov G, Plaseska-Karanfilska D (2009) Y Chromosome single nucleotide polymorphisms typing by SNaPshot minisequencing. Balkan Journal of Medical Genetics 12 (2).

[33] Plaseski T, Noveski P, Popeska Z, Efremov GD, Plaseska-Karanfilska D (2012) Association study of single-nucleotide polymorphisms in FASLG, JMJDIA, LOC203413, TEX15, BRDT, OR2W3, INSR, and TAS2R38 genes with male infertility. J Androl 33: 675–683. 22016351 10.2164/jandrol.111.013995

[34] Madjunkova S, Volk M, Peterlin B, Plaseska-Karanfilska D (2012) Detection of thrombophilic mutations related to spontaneous abortions by a multiplex SNaPshot method. Genet Test Mol Biomarkers 16: 259–264. 22023244 10.1089/gtmb.2011.0173 PMC3326265

[35] Atanasovska B, Bozhinovski G, Plaseska-Karanfilska D, Chakalova L (2012) Efficient detection of Mediterranean beta-thalassemia mutations by multiplex single-nucleotide primer extension. PLoS One 7: e48167. 23110203 10.1371/journal.pone.0048167 PMC3482202

[36] Untergasser A, Cutcutache I, Koressaar T, Ye J, Faircloth BC, et al (2012) Primer3–new capabilities and interfaces. Nucleic Acids Res 40: e115. 22730293 10.1093/nar/gks596 PMC3424584

[37] Costa C, Costa JM, Martin J, Boissier B, Goossens M, et al (2008) Multiplex allele-specific fluorescent PCR for haplotyping the IVS8 (TG)m(T)n locus in the CFTR gene. Clin Chem 54: 1564–1567. 18755906 10.1373/clinchem.2008.103259 PMC4710781

[38] Cuppens H, Lin W, Jaspers M, Costes B, Teng H, et al (1998) Polyvariant mutant cystic fibrosis transmembrane conductance regulator genes. The polymorphic (Tg)m locus explains the partial penetrance of the T5 polymorphism as a disease mutation. J Clin Invest 101: 487–496. 9435322 10.1172/JCI639 PMC508589

[39] Groman JD, Hefferon TW, Casals T, Bassas L, Estivill X, et al (2004) Variation in a repeat sequence determines whether a common variant of the cystic fibrosis transmembrane conductance regulator gene is pathogenic or benign. Am J Hum Genet 74: 176–179. 14685937 10.1086/381001 PMC1181905

[40] Patrizio P, Asch RH, Handelin B, Silber SJ (1993) Aetiology of congenital absence of vas deferens: genetic study of three generations. Hum Reprod 8: 215–220. 8473422 10.1093/oxfordjournals.humrep.a138025

[41] Bustamante-Aragones A, Gallego-Merlo J, Trujillo-Tiebas MJ, de Alba MR, Gonzalez-Gonzalez C, et al (2008) New strategy for the prenatal detection/exclusion of paternal cystic fibrosis mutations in maternal plasma. J Cyst Fibros 7: 505–510. 18573697 10.1016/j.jcf.2008.05.006

